# Water Resource Planning Under Future Climate and Socioeconomic Uncertainty in the Cauvery River Basin in Karnataka, India

**DOI:** 10.1002/2017WR020970

**Published:** 2018-02-03

**Authors:** Ajay Gajanan Bhave, Declan Conway, Suraje Dessai, David A. Stainforth

**Affiliations:** ^1^ London School of Economics and Political Science Grantham Research Institute on Climate Change and the Environment London UK; ^2^ Sustainability Research Institute and ESRC Centre for Climate Change Economics and Policy, School of Earth and Environment University of Leeds Leeds UK; ^3^ London School of Economics and Political Science Centre for the Analysis of Time Series London UK; ^4^ Department of Physics University of Warwick Coventry UK

**Keywords:** India, climate change, socioeconomic change, narratives, WEAP, adaptation pathways

## Abstract

Decision‐Making Under Uncertainty (DMUU) approaches have been less utilized in developing countries than developed countries for water resources contexts. High climate vulnerability and rapid socioeconomic change often characterize developing country contexts, making DMUU approaches relevant. We develop an iterative multi‐method DMUU approach, including scenario generation, coproduction with stakeholders and water resources modeling. We apply this approach to explore the robustness of adaptation options and pathways against future climate and socioeconomic uncertainties in the Cauvery River Basin in Karnataka, India. A water resources model is calibrated and validated satisfactorily using observed streamflow. Plausible future changes in Indian Summer Monsoon (ISM) precipitation and water demand are used to drive simulations of water resources from 2021 to 2055. Two stakeholder‐identified decision‐critical metrics are examined: a basin‐wide metric comprising legal instream flow requirements for the downstream state of Tamil Nadu, and a local metric comprising water supply reliability to Bangalore city. In model simulations, the ability to satisfy these performance metrics without adaptation is reduced under almost all scenarios. Implementing adaptation options can partially offset the negative impacts of change. Sequencing of options according to stakeholder priorities into Adaptation Pathways affects metric satisfaction. Early focus on agricultural demand management improves the robustness of pathways but trade‐offs emerge between intrabasin and basin‐wide water availability. We demonstrate that the fine balance between water availability and demand is vulnerable to future changes and uncertainty. Despite current and long‐term planning challenges, stakeholders in developing countries may engage meaningfully in coproduction approaches for adaptation decision‐making under deep uncertainty.

## Key Points

- An iterative approach combining qualitative and quantitative methods is used to assess robustness of adaptation options and pathways
- Performance criteria for the Cauvery River Basin in Karnataka are not satisfied under almost all scenarios with or without adaptation
- A coproduction approach helps target stakeholder priorities in the hydrological modeling and design of long‐term adaptation pathways

## 1 Introduction

In many developing countries, climate change is happening alongside rapid socioeconomic change. Uncertainty about the rate and extent of change makes water resources planning challenging. Conflicting interests make decision‐making a challenging process (Thissen et al., 2017), exacerbated by differences in the applicability and effectiveness of single or portfolios of response options. A range of approaches, broadly categorized as Decision‐Making Under Uncertainty (DMUU), seek to address this challenge, particularly for climate change adaptation. DMUU approaches, which include robust decision‐making, information‐gap decision theory, decision scaling, and dynamic adaptive pathways (Herman et al., 2015), find their basis in the principle that “assess‐risk‐of‐policy” is better than a “predict‐then‐act” approach, under conditions of deep uncertainty (Walker et al., 2013). These methods often combine modeling‐based assessments with stakeholder engagement. Indeed, stakeholder engagement is crucial for understanding basin management and decision‐making contexts, stakeholder priorities and perceptions, ongoing initiatives, future options, and decision‐specific characteristics (Hallegatte, 2014).

Interdisciplinary and knowledge coproduction approaches, which include social and biophysical dimensions, are increasingly recognized as critical to support water resources decision‐making and fulfill stakeholder requirements (Brown et al., 2015; Fant et al., 2016; Lund, 2015; Sivapalan et al., 2012; Troy et al., 2015; Vogel et al., 2015; Wheater & Gober, 2015). Applying DMUU approaches for water resources planning in developing countries is particularly challenging because of limited or inaccessible observations, poorly regulated water systems, ineffective legislation, and water governance mechanisms (Bhave et al., 2016a). Nevertheless, the uncertainties associated with rapid socioeconomic development and future climate change underscore the potential usefulness of integrated DMUU approaches for water supply and demand planning in developing countries (Dessai & Wilby, 2011; Lempert & Kalra, 2011). Developing country applications are, however, limited. Apart from a study on Lima's (Peru) water resources planning (Kalra et al., 2015) and flood risk management in Ho Chi Minh City (Vietnam) (Lempert et al., 2013), there are no other documented studies in developing countries. As the global adaptation agenda moves to implementation, there is an urgent need to build the evidence base for applications of DMUU approaches in developing country decision contexts.

Here we apply a DMUU approach in the Cauvery River Basin in Karnataka (CRBK) in India which exemplifies two key sources of uncertainty about the future: regional climate change and the effects of rapid socioeconomic development. We develop an iterative multi‐method approach, linking qualitative (stakeholder‐based) and quantitative (modeling‐based) analysis, that is consistent with coproduction thinking (Clark et al., 2016). The motivation is to examine the transferability of DMUU approaches, largely developed in advanced economies, to a developing country context. Specifically, we address the following research aims:

- To develop and apply an iterative methodology that combines expert judgment, stakeholder engagement and model‐based water resource assessments for DMUU.
- To assess how plausible scenarios of future climate and socioeconomic development affect water resources in the CRBK.
- To examine the extent to which individual options and portfolios of sequenced options—referred to as Adaptation Pathways—can address local and basin‐wide performance criteria.

The paper is divided into five sections. In section 2, we describe the study area and the decision context and section 3 describes the qualitative and quantitative methods. Section 4 presents the results, and in section 5, we discuss these results and future research directions.

## 2 Study Area

### 2.1 Sociopolitical Context

The Cauvery River Basin (CRB) is shared between the riparian states of Karnataka, Tamil Nadu, Kerala, and Puducherry. Historical, political, and socioeconomic factors have contributed to a decades‐long conflict over water resources between the riparian states (Sivakumar, 2011; Vanham et al., 2011). India's National Water Policy (Government of India, 2012) supports integrated water resources management, but falls short of providing specific measures because legal frameworks and their implementation are state responsibilities. Political differences hinder effective national‐state coordination, while state‐level administration of water resources is fragmented, with different competencies and unwillingness to share information (Ferdin et al., 2010). Policy‐making in the CRB has been in constant flux, with ad‐hoc and changing water resource allocations, culminating in the Cauvery Water Disputes Tribunal (CWDT) (2007) allocation. This tribunal allocated water flows in the Cauvery river to each state, herein referred to as the “instream flow requirement” (IFR). These allocations were based on an assessment that with “50% dependability” (which intrinsically accounts for interannual variability, but not longer‐term changes) there should be ∼21 billion m^3^ of annual water availability. The main upstream state, Karnataka (∼41% of CRB area), was allocated 36.4% of this amount, while downstream Tamil Nadu (∼54% of the area) was allocated 56.6% (CWDT, 2007). Karnataka and Tamil Nadu continue to contest this allocation in the Supreme Court (Sivakumar, 2011). This ongoing interstate conflict for water resources limited our ability to engage with as many relevant stakeholders as desirable.

### 2.2 Cauvery River Basin Within Karnataka (CRBK)

**Figure 1:**
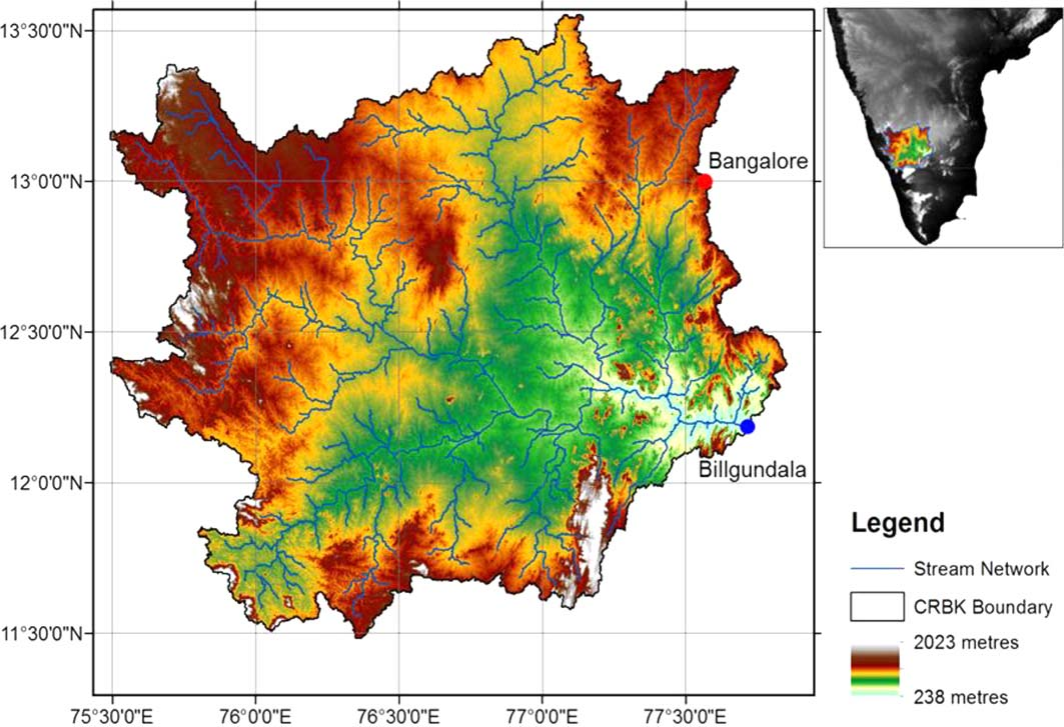
The Cauvery River Basin in Karnataka (CRBK) location, topography, and stream network developed using the Advanced Spaceborne Thermal Emission and Reflection Radiometer (ASTER) Digital Elevation Model (DEM). The Western Ghats form the western ridge of the basin, whereas Bangalore is located on the north‐eastern ridge (red dot). The river flows in a south‐easterly direction toward downstream Tamil Nadu, and the outlet of the CRBK is Billgundala (blue dot).

The Cauvery River Basin within Karnataka (CRBK) is a subbasin of the CRB, located within Karnataka (∼35,960 sq. km), defined as the basin upstream of Billgundala (see Figure 1), the point where the river flows from Karnataka to Tamil Nadu. The CRBK is large and diverse, with considerable variations in precipitation, water availability, sensitivity of agriculture, adaptive capacity, and overall climate change vulnerability (Kumar et al., 2016). The Government of Karnataka (2002) advocates a basin‐scale, multisectoral approach for managing surface and groundwater resources. Multiple agencies are involved in water allocation and use within the CRBK, however, there are no institutional arrangements for interstate water management (State Water Policy of the Government of Karnataka, 2002). In this interstate context, Billgundala is, therefore, a key gauging station. It is monitored by the Central Water Commission (CWC) and long‐term flow data are publicly available. Monthly flows are used to determine whether Tamil Nadu receives the correct water allocation.

### 2.3 CRBK Water Availability and Demand

The mean basin‐wide annual total precipitation ranges from ∼900 to ∼2,000 mm (for the period 1971–2004) and values vary considerably across the basin. Long‐term precipitation indicates a modest decreasing trend, most prominent in the Western Ghats (Ministry of Water Resources, 2014), the area of highest precipitation and contribution to streamflow. Five key irrigated CRBK districts are Bangalore, Mandya, Mysore, Tumkur, and Hassan. Their total irrigated area has increased from ∼21% to ∼33% between 1985 and 2005 (ICRISAT, 2015), thereby increasing agricultural water demand. Meanwhile, Bangalore, capital of Karnataka and an Information Technology hub, has witnessed population growth from 4.7 million (2001) to 8.5 million (2011) along with increasing per‐capita water demand. Water pumped up from the Cauvery River satisfies a large portion of this water demand, along with an uncertain level of supply from local groundwater.

## 3 Methods

**Figure 2:**
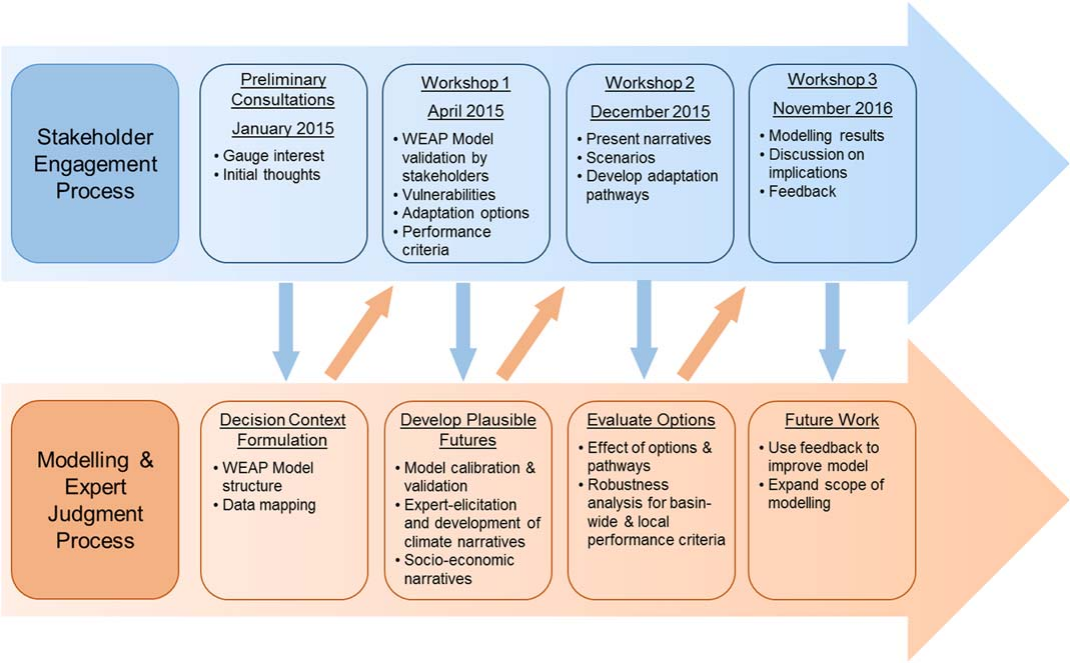
The iterative steps in the research design showing the sequencing of and linkages between qualitative (stakeholder engagement) and quantitative (modeling and expert judgment) methods.

We adopt an iterative approach which alternates between stakeholder engagement and modeling‐based analysis (Figure 2). We sought to enhance the credibility, legitimacy, and salience of the modeling process for stakeholders (following Cash et al., 2003) through a coproduction approach, whilst increasing our understanding of the decision‐making context. First, we investigated the decision contexts and gauged stakeholder interests followed by the development of a water resources model. We discussed this model with stakeholders (Workshop 1) to validate it for the CRBK. Stakeholder groups identified adaptation options and developed Adaptation Pathways for a worst‐case scenario combination of lower water availability and higher water demand (Workshop 2). Using the water resources model we evaluated the adaptation options and pathways against two decision‐critical performance metrics, followed by a feedback session in Workshop 3. In the following subsections, we describe the methods in detail.

This approach involved the following methodological innovations:

- Translation of qualitative climate and socioeconomic narratives into plausible quantitative scenarios of future precipitation and water demand.
- The use of stakeholder's expert judgment to identify adaptation options and construct Adaptation Pathways.
- Exploration of the robustness of adaptation options and pathways against different performance metrics under various combinations of future climatic and socioeconomic changes.

### 3.1 Qualitative Methods

#### 3.1.1 Stakeholder Engagement in the CRBK

We first identified key stakeholders across water resources management, urban planning, agriculture, and the environment sectors by consulting with academics and researchers (see supporting information Table S4 for stakeholder affiliations and responsibilities). Subsequent consultations and workshops were held with slightly different combinations of stakeholders (affected by availability) including academics, nongovernmental organization representatives, Karnataka water resource decision‐makers, Bangalore water board decision‐makers, Bangalore urban planners, and Karnataka agriculture department decision‐makers. Preliminary consultations (January 2015) with 15 stakeholders and experts took the form of informal one‐to‐one meetings, to understand the water resource decision contexts, vulnerabilities, range of adaptation options, and performance metrics, and hence align the research with key urban and basin‐wide plans. These interactions facilitated characterization and spatial disaggregation of baseline water availability and water demand by agriculture and urban areas. The consultations also helped us to communicate our research focus to stakeholders, gauge their interest (some were concerned in being involved because of the ongoing hydro‐political issues between Karnataka and Tamil Nadu), and gain their trust. The first participatory workshop (April 2015) focused on developing a shared understanding of the decision context and eliciting key aspects of the basin; including specific river basin functions, system components, and interactions, and their relevance for the preliminary water resources model structure (presented at the workshop). Key basin vulnerabilities, along with adaptation options and performance metrics, were also identified and prioritized through a group discussion.

**Figure 3:**
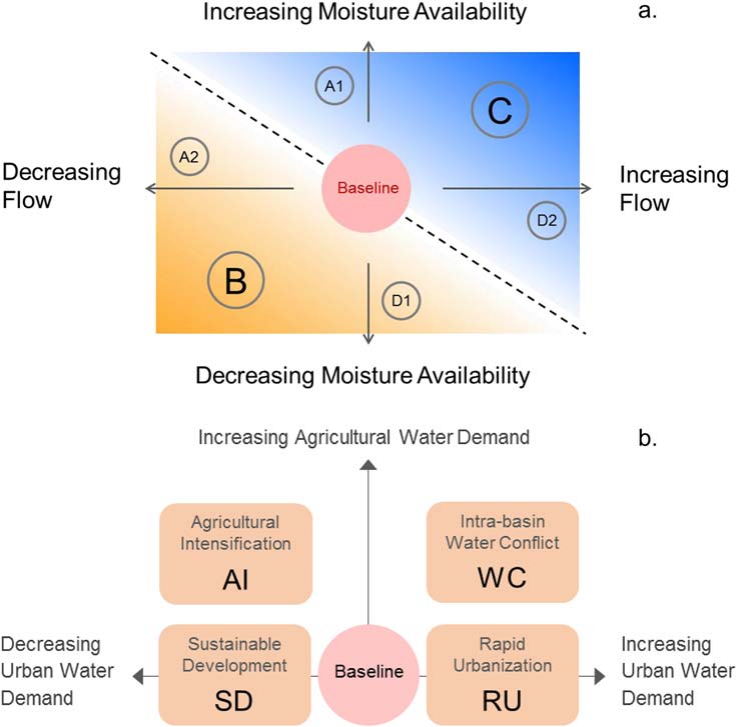
(a) Climate and (b) socioeconomic narratives for the CRBK for the 2050s. Climate narratives are displayed as a function of two key drivers (during the peak summer monsoon season): moisture availability in the Arabian Sea and strength of flow (West to East). The red circle in the center indicates the present day baseline conditions. Dashes divide the narratives into two triangles based on expected precipitation change. Upper (blue) triangle (covering narrative C) indicates increasing precipitation. Lower (brown) triangle (covering narrative B) indicates decreasing precipitation. Narratives A and D have precipitation similar to baseline conditions. Within A and D, A1 and D2 have a small increase in precipitation, while A2 and D1 have a small decrease in precipitation. Socioeconomic narratives that drive water demand are formulated as a function of agricultural and urban water demand and named according to their principal characteristic. Baseline conditions indicate present day demand.

Workshop 2 (December 2015) focused on developing Adaptation Pathways using the adaptation options identified in Workshop 1. We discussed the key assumptions which would be used for characterizing each adaptation option, which the stakeholders found acceptable. We presented newly derived qualitative climate narratives developed with climate scientists (Figure 3a, Dessai et al., 2018), and their quantified implications in terms of future precipitation changes (see section 3.2.1). We then presented socioeconomic narratives based on plausible future urban and agricultural water demand changes (Figure 3b). Finally, we presented a matrix of Narrative Combinations that spanned the largest uncertainty range (but not all combinations), consisting of five future climate narratives and three socioeconomic narratives (Table 1).

**Table 1:** Narrative Combinations Obtained From Combining Climate and Socioeconomic Narratives (Figure 3), Including Baseline Climate and Baseline Water Demand Conditions

Climate narratives (precipitation changes)	Socioeconomic narratives		
	Intrabasin water conflict (increasing water demand) denoted by D+	Sustainable development (decreasing water demand) denoted by D−	Baseline demand (no change in future water demand) denoted by D∼
C (large increase)	P++D+	P++D−	P++D∼
B (large decrease)	P−−D+	P−−D−	P−−D∼
A1 (small increase)	P+D+	P+D−	P+D∼
D1 (small decrease)	P−D+	P−D−	P−D∼
Baseline (no change)	P∼D+	P∼D−	P∼D∼

*Note*. Four changes in mean precipitation are defined and a baseline (no change in precipitation). Two socioeconomic narratives are used; Sustainable Development (SD) and Intrabasin Water Conflict (WC) and a baseline demand (no change in future water demand).

Due to time constraints only the most challenging Narrative Combination in terms of water availability was considered for sequencing adaptation options. This Narrative Combination consisted of the largest decrease in precipitation (P− −) and the largest source of rising demand (increasing urban and agricultural demand, D+). Participants were separated into groups with similar (where possible) responsibilities or experience in state government decision‐making, agriculture, industry, and water management. Some stakeholder groups were represented by individuals who understood much about the sector, but were not officially part of it; including retired civil servants, academics, and researchers. Also, depending on availability, some stakeholder groups had more individuals than others. Each group was asked to sequence the adaptation options based on their priorities in 5 year intervals from 2015 to 2055: we refer to these sequences as “Adaptation Pathways” (cf. Haasnoot et al., 2012). Hence, four Adaptation Pathways were obtained; Agriculture Stakeholders, Government Decision‐Makers, Industry Stakeholders, and Water Board. In Workshop 3 (November 2016), we presented and discussed modeling results with stakeholders to elicit feedback on communication of results.

#### 3.1.2 Climate and Socioeconomic Narratives

Uncertainty about the evolution of the Indian Summer Monsoon (ISM) under climate change is high (Turner & Annamalai, 2012) so instead of using projections from climate models we used a suite of climate narratives derived from an expert‐elicitation procedure as part of a complementary research exercise (Dessai et al., 2018) (see also Thompson et al., 2016). These narratives describe plausible changes in ISM precipitation in the Western Ghats region of the CRBK. Figure 3a shows the two main drivers identified by experts as the most important in influencing future precipitation changes in the CRBK: moisture availability over the Arabian Sea and strength of (wind) flow perpendicular to the Western Ghats. The product of the two drivers was defined as moisture flux, and experts suggested that changes in moisture flux lead to change in precipitation.

To characterize socioeconomic changes in the CRBK, we drew upon relevant literature (Alcamo et al., 2007; Arnell, 2004; Harrison et al., 2015; Kriegler et al., 2012) and the two principal water “users”—agriculture and urban areas. We used the two “users” to generate plausible trajectories of water demand change; we then characterized them and validated them with stakeholders (Workshop 2). Changes in urban demand depend on growing population and per‐capita demand, while changes in agricultural demand depend on changes in irrigated area. This led to four socioeconomic narratives, presented in Figure 3b (also supporting information Figure S1), which were discussed with stakeholders in Workshop 2 (see above). Since a future decrease in irrigated area was not considered plausible, the *Y* axis (Figure 3b) does not have a negative dimension (irrigated area does not reduce compared to the baseline). In order to keep the number of combinations manageable but still cover the range of plausible future water demand, only “Intrabasin Water Conflict” and “Sustainable Development” narratives were used. The climate and socioeconomic narratives were combined to generate “Narrative Combinations” of future conditions (Table 1). To enable a comparison of the sensitivity of the system to future precipitation and demand changes, scenarios with baseline precipitation (i.e., current conditions; 1983–2011) and baseline water demand (i.e., current conditions including potential evapotranspiration; 1983–2011) were also developed. Table 1 presents the 15 Narrative Combinations, including the overall baseline P∼D∼ Narrative Combination.

### 3.2 Quantitative Methods

#### 3.2.1 Converting Qualitative Narratives to Quantitative Precipitation Changes

Quantitative time series of future precipitation changes consistent with the qualitative narratives were generated to drive the water resources model. Dessai et al. (2018) estimated a relationship between moisture flux (product of the two drivers: moisture availability and strength of flow) (Figure 3a) and observed summer monsoon precipitation on the leeward side of the Western Ghats (CRBK) using ERA Interim (Simmons et al., 2007) and observational data. This relationship: y = 434.8x − 43.95, where x is the moisture flux (m s^−1^) and y is the precipitation (mm d^−1^), was used to generate a quantitative time series of future precipitation. We first perturbed moisture flux values by applying change factors to both drivers: moisture availability and strength of flow. For narratives B and C, we applied a ±10% change factor to both drivers to obtain four sets of changes in moisture flux that were perceived by the experts to be plausible. Narratives A and D are split into A1, A2 and D1, D2, where one driver is more influential than the other. Hence, we applied a ±10% change factor for the more influential driver, and a ±5% change factor for the other driver. Since the resultant precipitation change for the pairs A1 and D2, and D1 and A2 is identical, we use narratives A1 and D1 for further analysis. This led to four distinct changes in future mean precipitation for use in the WEAP analysis: narratives C (P++), B (P−−), A1 (P+), and D1 (P−). The narratives provided no information on changes in precipitation variability as the experts thought changes in interannual variability were too uncertain to estimate (see section 5 for a discussion of this limitation).

We derived a 50 year precipitation time series from 2006 to 2055 for the four changes in mean annual precipitation by assuming a linear rate of change from the average baseline precipitation (1981–2005) to the estimated future mean precipitation. Incremental changes at a monthly time step were applied to a twice repeated time series of the 25 year observed precipitation (observations were available for 1981–2005 from the Global Precipitation Climatology Centre; Schneider et al., 2008). The changes in precipitation were assumed to apply equally to the whole of the CRBK (the expert elicitation for change in the ISM focused on precipitation in the Western Ghats). As there is a significant correlation between precipitation in the Western Ghats and areas further east (Non‐Western Ghats area) in the CRBK (r = 0.70), the same approach was used to estimate precipitation in this Non‐Western Ghats area.

#### 3.2.2 Water Resource Modeling

The Water Evaluation And Planning (WEAP) model includes a dynamically integrated rainfall‐runoff hydrology module, based on the water balance accounting principle (Yates et al., 2005b, 2005a). It provides a flexible modeling environment for incorporating different water resources management options, water demand characteristics, allocation priorities, and scenario analyses, making it useful for evaluating the effectiveness of adaptation options and supporting decision‐making (Bhave et al., 2014; Lempert & Groves, 2010; Yates et al., 2015). We used the Food and Agriculture Organization (FAO) rainfall‐runoff method (Allen et al., 1998) which uses crop coefficients (Kc) for crops and other land uses and reference evapotranspiration (ETo, Penman‐Monteith) to determine crop evapotranspiration. Rainfall not consumed by ET is simulated as runoff and/or flow to groundwater. The parameters for calculating ETo were obtained from the ERA Interim reanalysis data with the exception of precipitation which came from the Global Precipitation Climatology Centre (supporting information Table S1). We used a monthly time step for calibration, validation, and future simulation motivated by: (i) the temporal resolution of the available hydrological and meteorological data, (ii) the legal stipulation of monthly flow requirement for Tamil Nadu by the CWDT award, and (iii) the residence time of the river basin.

**Figure 4:**
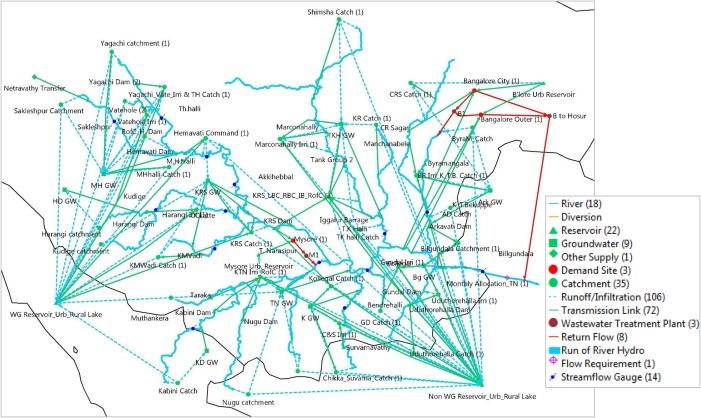
WEAP schematic for the CRBK. The schematic includes all items in the legend except “Run of River Hydro.” CRBK basin outflow is at Billgundala (used for WEAP calibration and validation) where it flows to downstream Tamil Nadu state. WEAP catchments were formulated in accordance with modeling requirements; reservoir location (see supporting information Table S2 for details), streamflow gauge location, location of irrigated areas, demand sites, and characteristics of adaptation options. Table 2 presents the main assumptions used to convert the adaptation options into water volumes.

Calibration (1983–1997) and validation (1998–2011) focused on the simulation of monthly and annual flows at the most downstream gauging station in Karnataka, Billgundala. Other gauging stations were used to improve the modeling of respective subcatchments (see below), but not for formal calibration and validation because data were insufficient and/or discontinuous. Figure 4 shows how the main subcatchments and sources of supply and demand in the basin were conceptualized. The FAO rainfall‐runoff method in WEAP has two parameters: crop coefficient (Kc) and effective precipitation. The range of monthly Kc values is based on Allen et al. (1998) for two crop cycles; Kharif (monsoon May/June–August) and Rabi (post‐monsoon September–December). Irrigated command areas of dams have Kc value ranges based on three cropping cycles. Monthly effective precipitation, which is the percentage of direct runoff, is calibrated based on topography, land‐use characteristics, and streamflow gauge data (wherever available) for each subcatchment. The simulation did not include groundwater‐surface water interactions and the effective precipitation parameter was adjusted to represent differences in irrigation intensity between catchments. Irrigation return flows were not explicitly modeled, but the response was lumped by the parameter effective precipitation. Here we assume that in irrigated areas (the majority of which is ponded rice and sugarcane) farmers restrict outflow by bunding the field (semipervious earthen wall).

In Workshop 1 (see Figure 2), stakeholders provided input for and validated the model structure to improve its ability to represent the natural and anthropogenic factors of the basin. Stakeholders pointed out that demand for irrigation in urban areas is often met by groundwater abstraction. Shallow dug wells and boreholes are used for abstraction, and although numerous, the extent and spatiotemporal distribution of abstraction is highly uncertain. Workshop participants were sceptical of the quality of publicly available information on groundwater levels. Groundwater was therefore modeled as a proportion of runoff from each subbasin. Both open dug wells and boreholes are used for supplementary irrigation, but information on these is often unavailable, so there is large uncertainty in the total quantity used. We therefore modeled groundwater in the nine intensely irrigated areas as a bucket which is replenished during the monsoon and stored water becomes available for satisfying irrigation demand in an unconstrained manner. Because groundwater is used for satisfying irrigation demand it was assumed that return flows were not generated. The storage capacity for each groundwater node was manually calibrated based on observed streamflow gauge data (available for different periods—see supporting information Figure S3) and observed reservoir levels.

The validated WEAP model was run for the 15 future Narrative Combinations (Table 1) with and without adaptation options. Ten Narrative Combinations in the future period include projected changes in potential evapotranspiration (ET) (see section 3.2.3), while five Narrative Combinations (D∼) do not include changes in future ET. Simulations without any adaptation are referred to as Business‐As‐Usual (BAU) scenarios and with adaptation are referred to as “Adaptation Scenarios.” For each Narrative Combination that includes adaptation, we ran separate simulations with each of the 17 individual adaptation options and then four simulations referred to as “Adaptation Pathways” in which all options were applied together but initiated at different points in the future. This resulted in 21 Adaptation Scenarios for each Narrative Combination (15), leading to a total of 315 Adaptation Scenarios (see Table 3).

**Table 2:** Assumptions Used to Quantify Stakeholder Identified Adaptation Options

Adaptation options		
13 stakeholder‐identified adaptation options (Workshop 1) were characterized and presented to stakeholders in Workshop 2. Two variants were developed for four options—urban rain/grey water harvesting, drip irrigation, and microirrigation which resulted in a total of 17 options. Their characteristics were converted into model‐relevant information. Each option was applied in January 2021 (in all Adaptation Scenarios), while for the Adaptation Pathways, the start‐up year followed the pathway‐specified sequence and timing (Figure 5). Mentioned below are the key assumptions for each adaptation option (with more detail in supporting information Figure S3). Options shaded grey (from 1 to 11) address water demand, while options shaded blue (12–17) address water supply issues.		
1	25% Urban Grey Water Recycling	• 25% reduction in annual water use rate and water consumption. • Increase urban water demand from 43.8 m^3^/yr (current estimate) to 54.75 m^3^/yr for scenarios with increased urban water demand.
2	50% Urban Grey Water Harvesting	• 50% reduction in annual water use rate and water consumption. • Increase urban water demand from 43.8 m^3^/yr (current estimate) to 54.75 m^3^/yr for scenarios with increased urban water demand.
3	25% Urban Rain Water Recycling	• Same as 25% Urban Grey Water Recycling.
4	50% Urban Rain Water Harvesting	• Same as 50% Urban Grey Water Recycling.
5	Better Enforcement of Laws	• 10% reduction in annual urban water demand and a reduction of water consumed in urban areas by 33%.
6	Urban Water Pricing	• We assume an indicative 50% reduction in annual urban water demand and reduction in water consumed from the current 30% to 20%.
7	1.5 mha Microirrigation	• 50% reduction in demand in 1.5 mha (million hectares) of irrigated area.
8	2.5 mha Microirrigation	• 50% reduction in demand in 2.5 mha (million hectares) of irrigated area.
9	5% Drip Irrigation	• Change irrigated fraction of historically irrigated catchments. • Water demand reduces by 80% for 5% of the irrigated area.
10	10% Drip Irrigation	• Change irrigated fraction of historically irrigated catchments. • Water demand reduces by 80% for 10% of the irrigated area.
11	Agricultural Water Pricing	• A 25% reduction in crop water demand assumed in the irrigated command areas of the four major reservoirs: Krishnaraj Sagar Dam, Harangi Dam, Hemavati Dam, and Kabini Dam.
12	Interbasin Transfer (100 MCM)	• Water transfer from neighboring Netravathy basin to Hemavathy basin (tributary of Cauvery) of 100 Million Cubic Meters (MCM).
13	Interbasin Transfer (88 MCM)	• Water transfer from neighboring Netravathy basin to Hemavathy basin (tributary of Cauvery) of 88 MCM.
14	Cauvery Stage V—Phase I	• 500 Million Liters per Day (MLD) supply from Cauvery river to Bangalore.
15	Cauvery Stage V—Phase II	• Extension of Phase I by a further 270 MLD.
16	Urban Lake Restoration	• Off‐stream reservoir with 210 million cubic meter (MCM) in Bangalore and 10 MCM in Mysore. • We divert all city runoff to reservoirs. Water available is used to satisfy urban water demand, while excess water runs off to the river.
17	Urban and Rural Lake Restoration	• Urban and rural reservoirs (200 lakes with a total capacity of 420 MCM). • 10% of the runoff from the respective catchments is diverted to the reservoirs, the water is used to satisfy irrigation water demand.

**Table 3:** *Summary of WEAP Model Runs, Incorporating 15 Narrative Combinations (Table 1), 21 Adaptation Scenarios (Table 2 for Adaptation Options and Figure 5 for Adaptation Pathways) and Business As Usual (BAU) Without Adaptation*

Narrative Combinations (N = 15)	Adaptation scenarios (N = 21)								Business As Usual (no adaptation (N = 1)
	Adaptation options (N = 17)				Adaptation pathways (N = 4)				
	1^a^	2	…	17	1^b^	2	3	4	
P∼D∼^c^									
P++D+									
P++D∼									
P++D−									
See Table 1 for full list									

a See Table 2 for explanation of all adaptation options.

b See Figure 5 that explains the four Adaptation Pathways identified by different stakeholder groups.

c Baseline; no climate change, no change in demand.

#### 3.2.3 Key Assumptions in the Water Resources Modeling

Precipitation: Qualitative climate narratives were translated into quantitative future precipitation for two distinct subregions of the CRBK: the Western Ghats and the Non‐Western Ghats. Precipitation time‐series were derived for all four climate narratives.

Evapotranspiration: Qualitative climate narratives did not include ET‐relevant information. We assumed a 1%/yr linear increase in potential ET for 10 Narrative Combinations with changing water demand (D+ and D−), which is consistent with model‐based findings in the region (Mishra et al., 2017). For five Narrative Combinations of baseline demand (D∼), we do not include changes in future ET to keep the water demand constant.

Irrigation: Irrigated area of districts (ICRISAT, 2015) (supporting information Figure S2) for the 1986–2005 period was used for linear extrapolation of trends to project future irrigation area. Irrigated area for scenarios with decreasing demand was held constant because large‐scale conversion of irrigated to nonirrigated land was assumed by stakeholders to be unlikely.

Urban Water Demand: Current and future water demand was estimated to be 120 lpcd (liters per capita/d), based on the Central Public Health and Environmental Engineering Organisation (2005) recommendations. Linear population projections for Bangalore and Mysore are used based on four census data points (1981, 1991, 2001, and 2011).

Table 3 summarizes the key aspects of the WEAP model runs, and provides directions to methodological details for them so that results in section 4 can be better understood, including Narrative Combinations and model runs with and without adaptation.

## 4 Results

### 4.1 Stakeholder Engagement and Adaptation Pathways

The most important insights from the stakeholder consultation process are summarized in Table 4. These are organized as key basin functions, priorities for water allocation, and critical vulnerabilities and drivers of change relevant for water resources in the basin. The adaptation options identified are listed in Table 2. Information from the stakeholders helped structure the research design and inform the model‐based analysis. Key functions of agricultural and urban water demand informed the development of socioeconomic drivers, while the dependence on precipitation in the Western Ghats informed development of the climate narratives. The adaptation options reveal the stakeholders' perspectives on what options are realistically available, given their appreciation of the local context. Options included infrastructural (supply‐side), water demand management (urban and agricultural), and policy options.

**Table 4:** The Main Results From Stakeholder Consultations Used to Inform the Research Design and Model Simulations

Key basin functions	Water allocation priorities	Key vulnerabilities and drivers
• Bangalore inner city and Bangalore outer city water supply. • Four major reservoir irrigated regions for sugarcane and paddy cultivation. • Power generation at Shivanasamudra project. • Industrial and commercial water requirements in urban areas.	During scarcity water supply is based on a hierarchy of priorities: (i) drinking water (ii) agriculture (iii) industry There is low priority for environmental flows. Tamil Nadu allocation includes environmental flows and outflows to the sea.	• Rapidly increasing population of Bangalore and rising demand. • Increasing irrigated area and increasing groundwater use for irrigation. • Shift to water intensive crops like sugarcane and paddy. • Dependence on high precipitation in the Western Ghats for water supply. • Climate change; in particular its consequences for extremes.

Adaptation Pathways (Figure 5) were derived for the most challenging Narrative Combination of decreasing precipitation and increasing water demand (P−−D+), to capture stakeholder views on responses to the most extreme combination of narratives. Agriculture stakeholders prioritized urban water pricing much earlier (2015) than agricultural water pricing (2050). The decision‐maker and water board groups focused on earlier implementation of options (all options by 2035) compared to the other two groups (some options beyond 2035). The Adaptation Pathways (Figure 5) show that not a single adaptation option was common to all groups by 2020, but by 2025 all groups selected rainwater harvesting (25%), drip irrigation (5%), and urban lake restoration.

**Figure 5:**
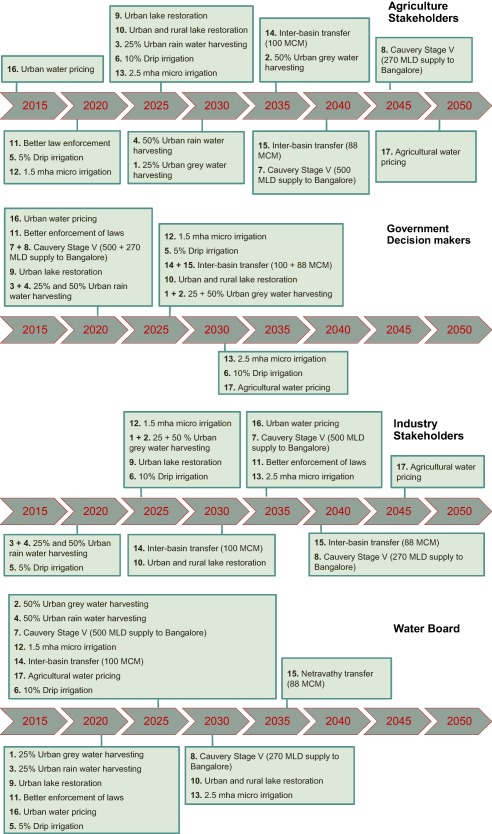
Schematic representation of Adaptation Pathways developed by the four stakeholder groups: Agriculture Stakeholders, Government Decision‐Makers, Industry Stakeholders and Water Board, during Workshop 2. Each pathway is a combination of the 17 adaptation options, and is based on each groups' preferences. The pathways were developed as a response to the critical Narrative Combination of decreasing precipitation and increasing urban and agricultural demand (P−−D+).

Two performance metrics were selected and agreed by all the groups to be the most important. One represents intrabasin considerations (Bangalore city demand coverage) and one captures basin‐wide issues (the IFR at Billgundala). We used the CWDT (2007) allocation as the basin‐wide performance metric, but we note that there is some uncertainty regarding the long‐term relevance of this allocation given the changing socioeconomic and political situation in Tamil Nadu and Karnataka. Furthermore, the allocation is unclear about how the concept of 50% dependability of water is applied, especially during relatively dry years. The verdict itself is being challenged by both states in the Supreme Court. The current water allocation was therefore taken only as a guiding metric for evaluating adaptation options.

An important feedback in Workshop 3 was that although the CWDT award provides the monthly downstream water requirement, it is essential to assess it against annual and 5 year total streamflow. The reasons for this included:

1. The presence of the large Mettur dam (1.5 billion cubic meter) immediately after the Cauvery enters Tamil Nadu which provides the state with an opportunity to buffer flows. This means that, to a certain extent, Tamil Nadu can absorb high flows and tolerate low flows.
2. The CWDT has determined the allocation at a 50% dependability level, which means that, by definition, for some years, downstream allocation cannot be satisfied. So a longer term performance assessment is appropriate.
3. The existence of other storage facilities, and the water use dynamics within the basin, mean that actual flows do not always meet the allocation on a month by month basis.

### 4.2 Water Resource Model Calibration and Validation

**Figure 6:**
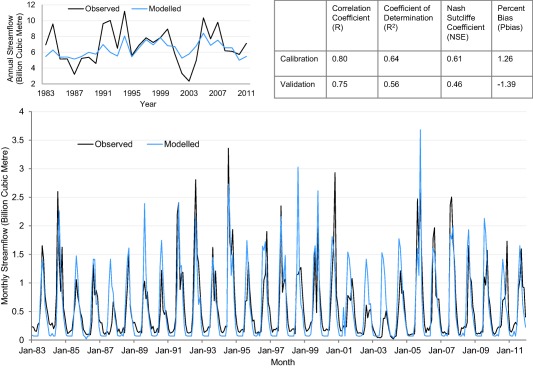
Observed and modeled streamflow at a monthly and annual (inset) time step at Billgundala for the WEAP model calibration (1983–1997) and validation (1998–2011) periods, and corresponding goodness of fit statistics for monthly streamflow.

In a complex and rapidly changing river basin, uncertainties arising from input data, water demand dynamics, changing reservoir operational rules, simplified representation of model components (such as groundwater and irrigated catchments), and the challenge of simulating rainfall‐runoff response in monsoon regions using WEAP (Bhave et al., 2014), all act to constrain model performance. Model calibration and validation for the last gauging station, Billgundala, indicates how these challenges manifest in terms of model ability to reproduce observed streamflow characteristics (Figure 6). While the model captured the large seasonal changes in streamflow reasonably well, it was less effective in simulating the interannual variability, particularly in the validation period (significant reduction in NSE value). This has implications for the interpretation of the future simulation results (see section 5). Nevertheless, the model performance is considered satisfactory for simulating long‐term changes in water availability (Moriasi et al., 2007; Ritter & Muñoz‐Carpena, 2013) and as a tool for exploring the sensitivity of streamflow to climate changes and the performance of adaptation measures.

### 4.3 Results of Future Simulations

We present results of the future simulations in terms of the two metrics: coverage of the instream flow requirement (IFR) at Billgundala and Bangalore city demand coverage.

#### 4.3.1 Basin‐Wide Metric: Instream Flow Requirement (IFR) at Billgundala

##### 4.3.1.1 Business As Usual (Scenarios With no Adaptation)

**Figure 7:**
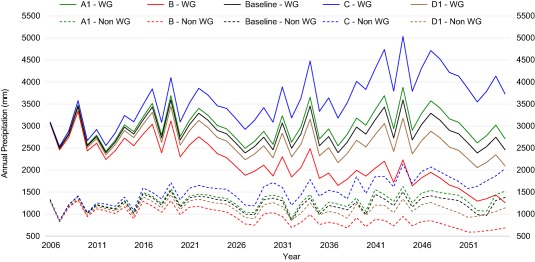
Future average annual precipitation of Western Ghats (WG, solid lines) and Non‐Western Ghats (Non‐WG, dashed lines) regions for five future climate narratives; baseline precipitation (baseline), small increase (A1), large increase (C), small decrease (D1) and large decrease (B).

Stakeholders highlighted that precipitation in the Western Ghats is the primary determinant of water availability in the CRBK. Western Ghats and Non‐Western Ghats precipitation time series (Figure 7) show the large difference in mean precipitation. The future annual precipitation time series for these regions, derived from the climate narratives, produced a very wide range of precipitation (about ±50%) by 2055. The series were used to drive WEAP model future simulations.

**Figure 8:**
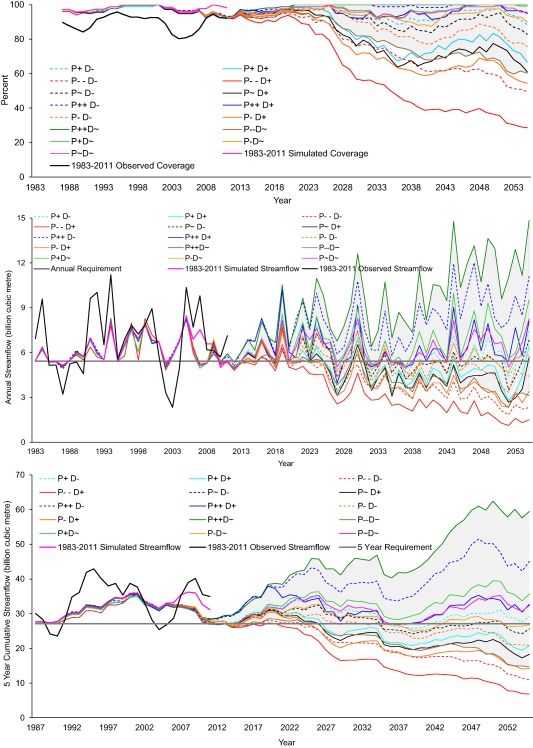
15 Business‐As‐Usual (BAU) scenarios and simulations against the basin‐wide metric. (top) The 5 year moving average of instream flow requirement (IFR) monthly coverage. Coverage is the percent of months in which the IFR for downstream Tamil Nadu is met, calculated over a 5 year sliding window period. Monthly coverage = min(100%, 100 × simulated instream flow/IFR). BAU simulations use observed precipitation from 1983 to 2005 followed by the scaled baseline precipitation (adjusted according to the climate narratives) from 2006 to 2055. (middle and bottom) Annual and 5 yearly streamflow simulations, respectively, for BAU scenarios at Billgundala gauging station. In each plot, bold black lines indicate observations (1983–2011), bold pink lines indicate simulations for observed period (1983–2011) and grey shaded region indicates the range for BAU scenarios with baseline water demand (see section 3.2.1). The pink line uses observations up to 2011 and thus deviates from the simulations for the period 2006–2011. The horizontal black lines in middle and lower plots represent the integrated IFR requirement on annual and 5 yearly timescales.

Future simulations for 15 BAU scenarios (without adaptation) for streamflow and coverage of the IFR for downstream Tamil Nadu reveal substantial differences (Figure 8). The top plot shows the 5 year moving average of IFR monthly coverage (percent of months when IFR is met), while the middle and bottom plots show the total annual and 5 year streamflow. P∼D∼ represents a reference simulation for comparing the effects of different combinations of precipitation and water demand change. Monthly coverage and streamflow from observations (1983–2011) (bold black lines) show the modeled results in the context of their ability to simulate these variables in the observed period (bold pink lines). Moreover, there are differences between model simulations for some time steps during the observed period, which arise in part due to surface runoff being rerouted through the distributed reservoirs added to the schematic. The shaded grey region depicts the range of future changes only due to changes in precipitation. The impact of demand changes can be isolated by focusing on scenarios with increasing and decreasing demand for the same climate narrative.

For monthly coverage, by 2055, in all cases except P++D−, there is an increase in the fraction of time that the IFR requirement is not met. The effects of the climate/demand scenarios on this metric are consistent with the direction and magnitude of the scenarios but some of the scenarios produce very large changes which highlights important sensitivities in the CRBK system. Even with increasing precipitation, the combination of increasing demand and natural decadal variability (as represented by past observations) could lead to reduced IFR coverage in the future. This is demonstrated by the difference between coverage for scenarios P+D+ and P+D−.

A key feedback from stakeholders during Workshop 3 was that there is flexibility in the way the performance metric is satisfied over time (see section 4.1). Consequently, we also evaluated the extent to which streamflow requirements are met over annual and 5 yearly time steps (middle and bottom plots in Figure 8). This further demonstrates the importance of decreasing demand for fulfilling the streamflow requirement. Except for P−−D−, all scenarios with decreasing demand show an ability to meet, or almost meet, the 5 yearly streamflow requirement all the way through to the end of the simulations. Indeed, we find that the integrated streamflow consequences of small decreases in precipitation can be offset by demand reduction, as also demonstrated by the comparable streamflow for P∼D∼ and P++D+. For scenario P++D+, the IFR is not met in a substantial (but not majority) number of years (annual timescale, Figure 8, middle plot) and just a few years on a 5 year timescale (Figure 8, lower plot). For P++D−, the IFR is almost 50% above the requirement for all years. This contrast highlights the important role that changing water demand has on the system's future performance. Significant difference between streamflow for P∼D∼ and P∼D− (also differences between P+D∼ and P+D−, P++D∼ and P++D−, P−D∼ and P−D−, and P−−D∼ and P−−D−) indicates the scale of potential impact of increasing demand and ET on the ability to meet the IFR.

##### 4.3.1.2 Adaptation Scenarios (Options and Pathways)

**Figure 9:**
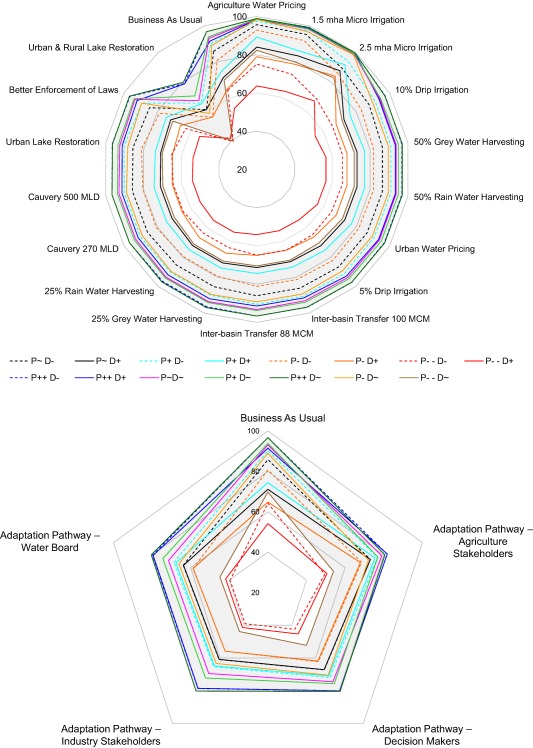
Reliability; percent of the monthly time steps in which the IFR was fully satisfied (upper) for each option separately and (lower) pathway for all 15 climate/demand Narrative Combinations (reliability calculated over the period 1983–2055). In the period 1983–2011 (calibration and validation), IFR reliability based on observed streamflow at Billgundala was 74.1%, while model simulated reliability is 90%. The grey shaded band represents the range of reliability across the Narrative Combinations with baseline demand (D∼).

Next we assess the ability of a range of adaptation options and pathways to satisfy the IFR performance metric across Narrative Combinations of future changes. Reliability, defined as the fraction of months in which IFR is fully satisfied, is used to compare the ability of options and pathways to meet the basin‐wide metric. Streamflow observations for the period 1983–2011, show an IFR reliability of 74%. The Baseline (P∼D+ and P∼D−) BAU climate scenarios (Figure 9) demonstrate the range of possible future IFR reliability ranging between 71% and 86%. Options and pathways show differences in their effectiveness (Figure 9).

The three most effective adaptation options all reduce agricultural water demand, which is the largest component of overall water use: 2.5 mha microirrigation produces a reliability >66%, 1.5 mha microirrigation which achieves >63%, and agriculture water pricing which gives reliability >63% across all scenarios. This suggests a need to consider trade‐offs between increasing microirrigation and decreasing overall water demand. For example, the additional effort or associated cost of increasing microirrigation from 1.5 to 2.5 mha (which achieves a 3% improvement in reliability) would need to be evaluated against changing future irrigation demand (for 1.5 mha the P−−D− scenario has 73% reliability cf. P−−D+ which has 63%). For most options, except microirrigation, agricultural water pricing and Urban and Rural Lake Restoration (URLR), reliability values are similar. As mentioned above (section 4.3.1.1), the scale of impact of future increase in ET is indicated by the difference in reliability for D∼ compared to D− Narrative Combinations.

Compared to the BAU scenarios, the option URLR significantly adversely affects the basin‐wide metric. This is primarily because the option acts as a distributed reservoir; it satisfies agricultural water demand but reduces runoff contribution. Although the total capacity is only comparable to similarly sized large reservoirs in the CRBK, in terms of basin outflow it is actively detrimental as an adaptation option (Figure 9). This property of URLR also affects the Adaptation Pathways, which all implement this option early in the sequence (Figure 5). The URLR shows similar reliability for the same precipitation regardless of demand changes, primarily because of the assumption that a proportion of catchment runoff is diverted to these distributed reservoirs. Results indicate that the dynamics of water availability and demand in the CRBK are finely balanced (Figure 7), and that further diversion of available runoff to agriculture could adversely affect the ability to meet the IFR. Understanding of this is reflected by the ongoing project for water transfer from the Netravathy basin into the CRBK (interbasin river transfer of 100 Mm^3^). Urban demand management options do not have a significant impact on the basin‐wide metric (compared to the BAU), suggesting that these options, although important from an urban perspective, are relatively insignificant in terms of volumetric impact compared to agricultural water demand options.

For any given scenario, the reliability of the different Adaptation Pathways is broadly similar but variations of about 10% are common (e.g., under P−D+ the Industry pathway has reliability of ∼56% while the Agriculture pathway has ∼68%) (Figure 9, lower plot). Since all pathways apply the same options, it is not surprising to find similarity in their performance. In most cases, the pathways do worse than the equivalent BAU simulation (where no adaptation measures are implemented), particularly for all the reduced demand scenarios. The reason is the negative impact of the URLR option which not only reduces reliability and overwhelms other adaptation options, but also undermines the effectiveness of demand reduction measures.

All pathways demonstrate low reliability for P−−D− and P−−D+ scenarios, while a cluster of scenarios lies around the 60–80% level. Although this level of reliability is similar to the observed reliability (74%) the model simulated reliability through the observed period (1983–2011) is 90% so all pathways under all scenarios indicate a greater than 10% reduction in future reliability. This is a worrying message for planners. The legal requirement on annual IFR has in fact been met for the entire 2007–2011 period (the period for which we have data since the CWDT ruling) but Figure 8 suggests that this recent high reliability is partly a result of a period that was favorable simply as a consequence of natural variability. The Agriculture Stakeholders Adaptation Pathway is generally more effective than the others. As Figure 5 indicates, agriculture stakeholders focus on addressing agricultural water demand and apply microirrigation earlier in the pathway than other stakeholders. This appears to be sufficient to improve overall reliability relative to the other pathways even though the others apply agricultural water pricing (the other most effective single option) earlier. The Industrial Stakeholders' pathway is marginally the least reliable under most scenarios; a likely consequence of applying microirrigation later than any of the others while also applying agricultural water pricing later than all but in the Agriculture Stakeholders' Adaptation Pathway.

#### 4.3.2 Local Metric (Bangalore City Demand Coverage)

**Figure 10:**
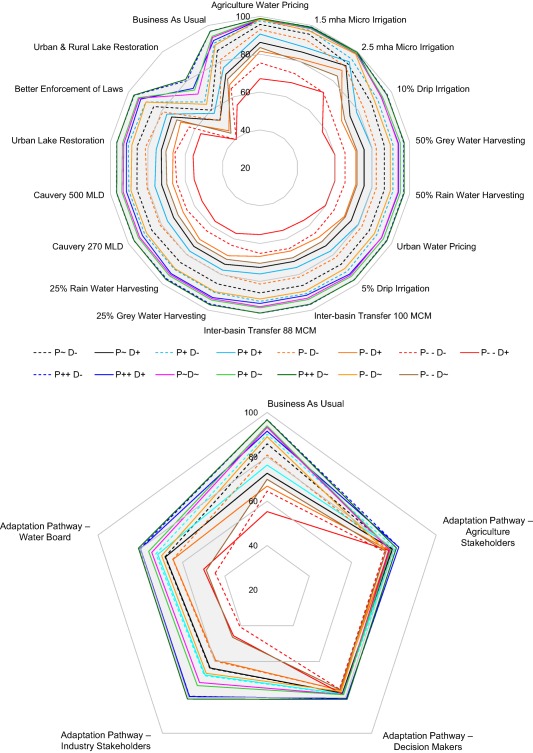
Reliability; the percent of monthly time steps in which water demand of Bangalore was fully satisfied (upper) for each option and (lower) pathway for all 15 Narrative Combinations. The grey shaded band represents the range of reliability across the Narrative Combinations with baseline demand (D∼).

Unmet water demand in Bangalore is critical because it is the administrative, economic and cultural hub of Karnataka state. Analyzing the reliability of options and pathways with regards to meeting Bangalore's water demand across all scenarios (Figure 10) provides insight regarding two issues. First, the upper plot depicts a pattern similar to the basin‐wide metric with the same reliability range (40–100%). This is understandable in the context of the system, because streamflow downstream of the last major dam is critical for both Bangalore's water supply and for meeting the IFR. This means that the ability to address the basin‐wide and the local metric are closely linked. In both cases, the dependence on precipitation is clear; as the stakeholders asserted, it is critically dependent on changes in precipitation in the Western Ghats. The modeling study thus corroborates the issues raised during stakeholder consultations. Second, the most effective individual option is 2.5 mha of microirrigation, while the most effective pathways (for both performance metrics) are those of agriculture stakeholders and government decision‐makers. Urban demand management options, particularly 50% grey/rain water harvesting and urban water pricing, have more impact on Bangalore's supply reliability, compared to the IFR reliability (Figure 9). However, apart from microirrigation and water pricing for agriculture, the other options have insufficient impact relative to the basin‐wide streamflow volumes, to make a big difference. It should be noted though that the stakeholder derived plausible characteristics for such adaptation options set a limit to their volumetric effects. Further iterations with stakeholders could potentially refine those characteristics in the light of these results. Since multiple options have a similar scale of benefits, their selection for implementation would depend on other criteria, such as economic feasibility, acceptability, and environmental benefits.

The results of Adaptation Pathways also demonstrate the dynamic between satisfying agricultural and urban demand. Government Decision‐Maker and Agriculture Stakeholder pathways have similar reliability for Bangalore. Government Decision‐Makers have preferred to address urban demand in the sequence compared to Agriculture Stakeholders, who focused on addressing agricultural demand first (Figure 5). Hence, while both pathways are equally robust for Bangalore, the focus on addressing agricultural demand helps the Agriculture Stakeholders pathway be more robust for IFR. Gradual and incremental agricultural demand management in the Industry Stakeholder pathway and delayed implementation of 1.5 mha/2.5 mha of microirrigation in the Water Board pathway affects the robustness of these pathways. The application of the URLR option across all the pathways provides more water within the CRBK for agriculture, but adversely impacts the IFR and Bangalore water supply. This suggests that there is a conflict between increasing water for agriculture and Bangalore, and the conflict between satisfying intrabasin demand (particularly agriculture) and the basin‐wide metric. This suggests that urban and agricultural water demand can, and should, be addressed in an integrated manner, based on a recognition of their interdependence and importance for addressing both local and basin‐wide metrics. Figure 10 demonstrates how large scale agricultural water demand management can, in this example, usefully address both the local and basin‐wide metrics. Similar to the basin‐wide metric, the URLR option adversely affects ability to satisfy urban water demand. This suggests that further water diversion to satisfy agricultural demand could disturb the existing fine balance between water availability and demand within and beyond the CRBK.

The direction and magnitude of future precipitation change in the CRBK are uncertain and so is the outcome of changes in urban and agricultural demand. Such deep uncertainty challenges adaptation decision‐making and infrastructure planning. Our results suggest that changes in both climatic and socioeconomic factors strongly influence critical indicators of future water resource system performance. Different Narrative Combinations have much greater impact than different adaptation options, i.e., uncertainty in climatic and social changes mostly overwhelm differences between the choice of adaptation options. The Adaptation Pathways sequenced by stakeholders encapsulate a range of streamflow responses that result from the application of adaptation options in different sequences. We observed a similar effect in all pathways, where high flows decrease, medium flows may decrease or increase, and low flows increase. Absorbing high flows is important in a monsoon‐dominated region (Bhave et al., 2016b) because it reduces the risk of downstream flooding, while increasing water availability during dry periods. High flows are absorbed because of a distributed reservoir formulation of the adaptation option URLR. This option reduces high flows from each subcatchment and provides additional water for satisfying urban/irrigation water demand. However, URLR also decreases streamflow to downstream Tamil Nadu, thus illustrating an important trade‐off between meeting local water demand and downstream water demand.

## 5 Discussion and Conclusions

In this study, we explore the robustness of adaptation options and pathways for a complex river basin, influenced by large and small reservoirs, and with ongoing competition for water at various spatial scales. This required customization and simplification of methods to suit the decision context, data constraints, and political sensitivities. We integrated stakeholder engagement and modeling in an iterative process, motivated by a desire to coproduce the work, and therefore increase the salience and the credibility of the results. We leveraged the relative advantages of qualitative and quantitative methods to enhance our ability to understand and model the system according to stakeholders' requirements.

Stakeholder judgments consider the various social, economic, political, and environmental criteria relevant to the identification of adaptation options and pathways. As the stakeholders identified Western Ghats precipitation as a crucial determinant of water availability, we developed climate narratives of plausible future ISM evolution in a complementary process (Dessai et al., 2018). Climate narratives coupled with socioeconomic narratives provided a range of plausible future conditions of water availability and demand. This aided long‐term thinking and the development of Adaptation Pathways which addressed stakeholder considerations about option types and timing of implementation. Eliciting feedback on results (Workshop 3) revealed their preferences for communicating results, including, comparing options and pathways separately, and discussing reliability results for annual and 5 year performance metrics together with monthly results.

It is important to note the limitations of this work. First, there was restricted access to some specific data, particularly observed rainfall. Second, the climate narratives, demand scenarios, and some aspects of the modeling were based on secondary information. Stakeholder/expert judgments inevitably introduce some subjectivity. The sporadic participation of some stakeholders and role‐playing may also have meant that stakeholder groups may not have been represented as accurately as real stakeholder groups. This is also the case for our estimation of the volumetric effects of the adaptation options, which, while based on evidence from similar interventions elsewhere, also incorporate the views of the CRBK stakeholders. The contested nature of some of these views was illustrated by a participant in Workshop 3 who questioned the assumption that the future irrigated area would be constant after implementing irrigation efficiency measures. It was suggested that in practice farmers would use water savings to expand the irrigated area, which may mean that a proportional reduction in demand may not occur (cf. Fishman et al., 2015). Third, stakeholder engagement in a river basin with significant tensions over water was challenging. The limited time available for workshops restricted the number of scenarios that could be considered and some evidence of stakeholder fatigue or restricted availability were factors in realizing stakeholder participation. Similarly, the expert elicitation focused only on precipitation change (behavior of the ISM); the consequences of warming for evapotranspiration rates and potential changes in variability were not considered.

Finally, using standard goodness of fit statistics, we considered the model performance was satisfactory for this type of study and given the complexity of the CRBK system and data scarcity. However, the model simulation of the annual and 5 year performance metrics was marginal (Figure 8). Stakeholders specifically requested these two performance metrics quite late in the study process (in Workshop 3), which limited the time available to calibrate these aspects of model performance. This is an important point from a DMUU research perspective; user‐defined bespoke performance metrics may be different to conventional criteria used in model assessment. Therefore, due consideration needs to be given to the simulation of such metrics and whether they are captured by conventional measures of goodness of fit. Limited data availability meant additional assumptions in the modeling. For instance, we model annually replenished groundwater because information on the extent of borehole‐based irrigation is unavailable and considered unreliable. The results of the future simulations incorporate important assumptions such as the characteristics of adaptation options (already noted), and the use of observed precipitation to characterize future precipitation variability. Noting also that the WEAP model was limited in its simulation of observed interannual variability (the basin response was dampened) we stress that our results are indicative, not definitive. Emphasis should be placed on the relative rather than the absolute magnitude of impacts.

The expert elicitation‐driven climate narratives provided a valuable means for exploring plausible future changes in decision relevant aspects which circumvented contentious issues of climate model reliability and the suitability of climate model‐based projections for this work. The narratives enhanced the stakeholders' understanding of the climate processes which drive Western Ghats precipitation change, and quantitative realizations aided the visualization of these changes. Exploring the robustness of adaptation options and pathways, against intrabasin and basin‐wide metrics helped workshop participants develop a more comprehensive understanding of the consequences of impacts and actions, particularly because of their end‐to‐end involvement in the research process. Crucially, the exploration of trade‐offs within and beyond the CRBK, could support adaptation decision‐making in managing the finely balanced dynamic between water availability and demand.

We demonstrate that there is complex interplay in the CRBK between precipitation, water demand from agriculture and urbanization, potential future ET, and implementation of various adaptation options. We also highlight uncertainties across the range of climatic, socioeconomic, and modeling‐related components which influences our ability (or inability) to gauge potential future changes, which could be explored through a more detailed sensitivity analysis. The time when specific thresholds of precipitation and streamflow are exceeded might change with resampled time series of precipitation variability and the use of climate model outputs for driving the water resources model. This case study demonstrates the uncertainties in defining and operationalizing performance criteria, as shown by the uncertain interpretation of the 50% reliability of flow in the CWDT, and the different timescales of applicability (monthly, annual and 5 yearly). We therefore interpret the results in light of these uncertainties and emphasize the relative differences between options and pathways, rather than absolute changes and thresholds.

Further exploration of uncertainties in economic and sociopolitical feedbacks; how social systems respond to changes in water availability (sociohydrological interactions), and water governance challenges (Srinivasan et al., 2016), would be very relevant in this region. Greater integration of uncertainties in, and interactions with, water‐related sectors such as, recreation, energy production, food security, rural livelihoods, and the natural environment would be valuable to other stakeholders. Future studies could also consider options not elicited from stakeholders in this study, such as groundwater management (laws, licenses, etc.) and cropping pattern changes. There are also opportunities for such DMUU research to draw insights from other research areas, such as collaborative modeling for complex systems (Langsdale et al., 2013), to enhance integration of modeling and participatory processes.

In conclusion, the deep uncertainty associated with future precipitation and demand for water in the CRBK is a critical feature of adaptation decision‐making and infrastructure planning. Our results show how changes in both climatic and socioeconomic factors strongly influence future water resource system performance. We found an iterative approach to DMUU valuable because it enabled the analysis to evolve to suit the decision context and stakeholder requirements. This facilitated stronger stakeholder engagement and feedback, an important constituent of knowledge coproduction.

## 6 Erratum

In Table 3 of the originally published version of this article, the term P∼D∼ was erroneously given as P∼D−. In the online HTML version of the article, Table 2 and Table 3 were transposed. These errors have been corrected and this may be considered the official version of record.

## Supporting information

Supporting Information S1
