# Supplementary material for: Water Resource Planning Under Future Climate and Socioeconomic Uncertainty in the Cauvery River Basin in Karnataka, India
Source: Water Resour Res. 2018 Feb 3;54(2):708–28. doi: 10.1002/2017WR020970 (PMC5900973; doi:10.1002/2017WR020970)
Supplement: Supplementary file 1 — Supporting Information S1 [file WRCR-54-708-s001.docx]

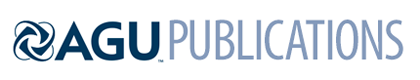


*Water Resources Research*

Supporting Information for

**Testing the waters in the developing world: Water resource planning under deep uncertainty in the Cauvery River Basin in Karnataka**

[Ajay Gajanan Bhave ^1 2^, Declan Conway ^1^, Suraje Dessai ^2^, David A. Stainforth ^1 3 4^]

^1^Grantham Research Institute on Climate Change and the Environment, London School of Economics and Political Science, London, WC2A 2AE, United Kingdom

^2^Sustainability Research Institute and ESRC Centre for Climate Change Economics and Policy, School of Earth and Environment, University of Leeds, Leeds, LS2 9JT, United Kingdom

^3^Centre for the Analysis of Time Series, London School of Economics and Political Science, London, WC2A 2AE, United Kingdom

^4^Department of Physics, University of Warwick, Coventry, CV4 7AL, United Kingdom

**Contents of this file**

Figures S1 to S3

Tables S1 to S4

**Introduction**

In Figure S1 we describe the socio-economic narratives. Using Figure S2 (a, b) we present data used to project future irrigation rates for socio-economic narratives. Figure S3 provides more information on streamflow data availability, and use for calibration and validation. Table S1 provides details of observed data used for modelling. Table S2 provides information on details of reservoirs used in the model development, and the relevant source. Table S3, provides detailed information on characteristics and assumptions used for the adaptation options. Table S4 provide details regarding the type, affiliation and roles/responsibilities of the stakeholders.


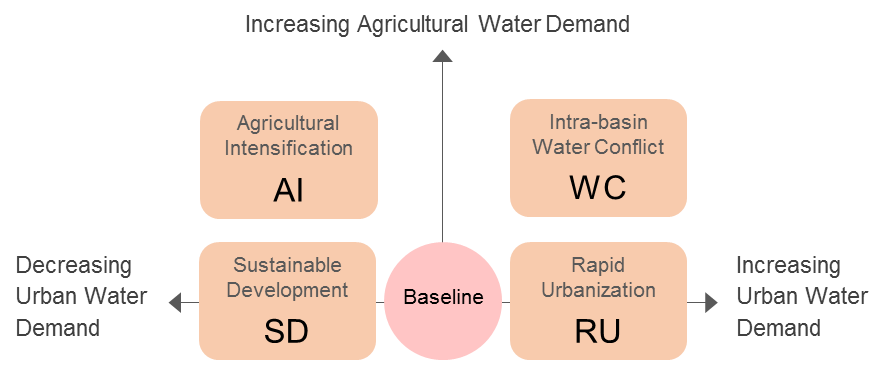


Figure S1. Socio-economic narratives developed for the Cauvery River Basin in Karnataka for the 2050s. Description of the figure below supplements information provided with Figure 3b in the article.

Sustainable Development: Principles of long-term sustainable water governance are legally adopted and implemented leading to reductions in water demand from agricultural and urban areas, including industrial and commercial users.

Agricultural Intensification: Expansion of agricultural land leads to pressure on forested regions while greater cropping intensity causes pressure on land resources. Greater groundwater extraction and greater irrigation water demand from reservoirs. Greater societal awareness regarding water and environmental protection issues leads to a decrease in urban water demand.

Rapid Urbanization: Agricultural water demand remains at baseline level. Larger and more populated urban areas, commercial development and increase in industrial water use lead to greater urban water demand.

Intra-basin Water Conflict: Simultaneous increase in water demand due to larger and more populated cities and expanding irrigated agriculture. Rapid and extensive groundwater extraction is observed in both urban and rural areas.

**Figure S2:** (a) Observed Average Irrigated Area (%) in the CRBK districts (b) Average irrigated area over time. Linear trend for 1986-2005 (black) was extrapolated to project future irrigated area for the socio-economic narrative ‘Intra-basin Water Conflict (WC). As mentioned in Table 3, for socio-economic narrative ‘Sustainable Development’ (SD) we hold the 2011 irrigation rates constant in the future.


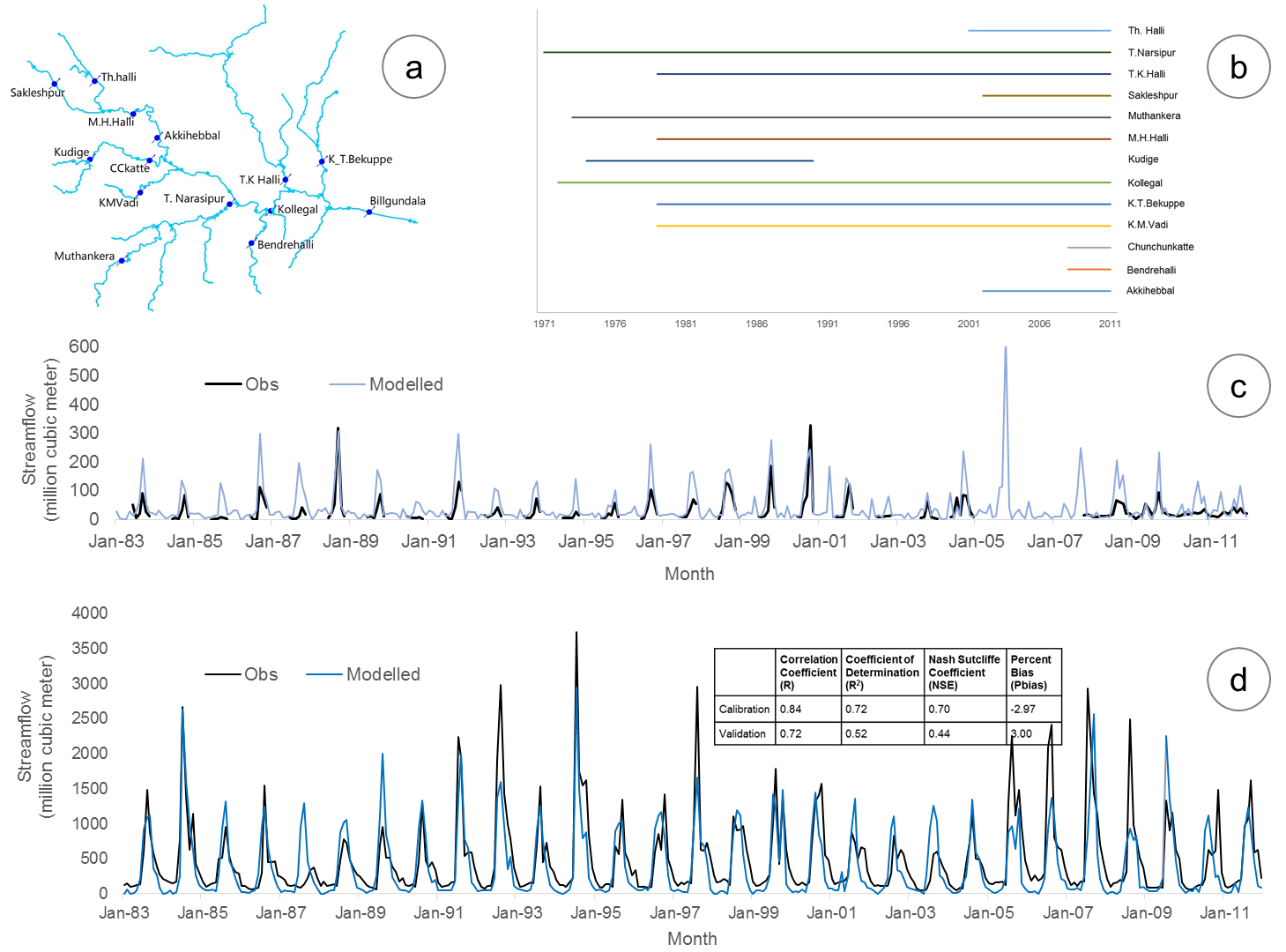


**Figure S3:** Key aspects of streamflow data used for calibration and validation. a) shows the distribution of gauging stations along the river network. b) depicts the length of data available for the gauging stations. c) depicts the observed and modelled streamflow for gauging station K.T.Bekuppe. It illustrates how data can be discontinuous although available for the calibration and validation period. d) depicts observed and modelled streamflow and goodness-of-fit statistics for Kollegal gauging station.

Different gauging stations provided information on different aspects, including sub-basin runoff response and reservoir characteristics (inflow / outflow / operation) of the four major reservoirs. Kollegal gauging station for instance is directly downstream of the last major dam in the CRBK, the Krishna Raj Sagar Dam. Streamflow response at this station was used to model reservoir operation.

The four most downstream gauging stations; Kollegal, K.T.Bekuppe, T.K.Halli and Billgundala, are critical to understanding the large scale streamflow response in the CRBK. Of these, continuous data for the period 1983-2011 is available only for Kollegal and Billgundala. The goodness-of-fit statistics for these two stations are comparable. Streamflow at Billgundala is critical for quantifying streamflow to downstream state Tamil Nadu, and hence formal calibration and validation was carried out for Billgundala.

| **Parameter** | **Data Period** | **Source** |
| --- | --- | --- |
| Precipitation | 1983 – 2011 | Global Precipitation Climatology Centre (Schneider et al. 2008) |
| Parameters for calculating Reference Evapotranspiration (ET) using Penman-Monteith method | 1983 – 2011 | ERA – Interim (Simmons et al. 2007) |
| Streamflow | Various individual periods between 1983 and 2011. | Ministry of Water Resources, India (2014) |
| Reservoir information | Various individual periods for 4 major reservoirs and 20 minor reservoirs | Water Resources Information System of India <http://india-wris.nrsc.gov.in/wris.html>  Department of Irrigation, Govt. of Karnataka <http://waterresources.kar.nic.in/projects.htm> |

Table S1 Observed climatological and hydrological data used for WEAP model development

| **Name** | **River** | **Districts benefitted** | **Irrigation potential (in thou. ha)** |
| --- | --- | --- | --- |
| Hemvathy Dam | Hemvathy | Hassan, Mandya, Tumkur | 283.6 |
| Kabini Dam | Kabini | Mysore | 87.9 |
| Krishnaraj Sagar Dam | Cauvery | Mandya, Mysore | 79.31 |
| Harangi Dam | Harangi | Kodagu, Hassan, Mysore | 54.59 |
| Nugu Dam | Nugu | Mysore | 10.53 |
| Votehole Dam | Votehole | Hassan | 7.48 |
| Uduthorehalla Dam | Uduthorehalla | Mysore | 6.48 |
| Arkavathy Dam | Arkavathy | Bangalore | 6.23 |
| Marconahally Dam | Shimsha | Tumkur | 5.94 |
| Taraka Dam | Cauvery | Mysore | 5.39 |
| Kama Samudra Lift Irrigation Scheme | Hemvathy | Hassan | 5.12 |
| Nanjapura Lift Irrigation Scheme | Cauvery | Mysore | 4.41 |
| Gundal Dam | Gundal | Mysore | 4.05 |
| Iggalur Dam | Shimsha | Bangalore, Mandya | 4.05 |
| Hutchana Koppalu Dam | Hemvathy | Hassan | 3.36 |
| Suvarnavathy Dam | Cauvery | Mysore | 2.83 |
| Kanva Dam | Kanva | Bangalore | 2.58 |
| Manchanbele | Arkavathy | Bangalore | 2.43 |
| Byramangala Dam | Vrishabhavathy | Bangalore | 2.23 |
| Chickkahole Dam | Chickkahole | Mysore | 1.65 |
| Nallur Amanikere Dam | Gundhu | Mysore | 1.3 |
| Hebbahalla Dam | Hebballa | Mysore | 1.21 |
| Chiklihole Dam | Chiklihole | Kodagu | 1.18 |
| Mangala Dam | Cauvery | Tumkur | 0.85 |
| **Total** | | | **584.7** |

Table S2. Characteristics of medium and large dams in the Cauvery River Basin in Karnataka as applied in our water resources modelling. Irrigation potential is conventionally assessed by the relevant state or central government bodies at the Detailed Project Report (DPR) development stage, and may also be termed ‘cultural command area’. This area is usually calculated using a fixed optimal/locally suitable cropping pattern (water demand) and analysis of water availability. Source: Water Resources Information System, Govt. of India (<http://india-wris.nrsc.gov.in/wrpinfo/index.php?title=Main_Page>)

| **Option** | **Unit change and Location** | **Description** |
| --- | --- | --- |
| Urban Grey Water Recycling – 25% | m^3^ / person per year  Urban location | Grey water recycling for Bangalore City, Bangalore Outer and Mysore. 25% reduction in annual water use rate and water consumption.  We increase urban water demand from 120 (43m^3^/year - current estimate) to 150 litres per day (54m^3^/year) for scenarios with increased urban water demand. Application of option reduces rate to 41m^3^/year. Consumption (amount that is lost from the system) is reduced by 25% to reflect the grey water reuse from 30% in current to 22.50%. |
| Urban Grey Water Recycling – 50% | m^3^ / person per year  Urban location | Grey water recycling for Bangalore City, Bangalore Outer and Mysore. 50% reduction in annual water use rate and water consumption.  We increase urban water demand from 120 (43.8m^3^/year - current estimate) to 150 litres per day (54.75m^3^/year) for scenarios with increased urban water demand. Application of option reduces rate to 27.375m^3^/year. Consumption (amount that is lost from the system) is reduced by 50% to reflect the grey water reuse from 30% in current to 15%. |
| Urban Rain Water Recycling – 25% | m^3^ / person per year  Urban location | Rain water recycling for Bangalore City, Bangalore Outer and Mysore. 25% reduction in annual water use rate and water consumption.  We increase urban water demand from 120 (43.8 m^3^/year - current estimate) to 150 litres per day (54.75m^3^/year) for scenarios with increased urban water demand. Application of option reduces rate to 41.0625m^3^/year. Consumption (amount that is lost from the system) is reduced by 25% to reflect rain water recycling from 30% in current to 22.50. |
| Urban Rain Water Recycling – 50% | m^3^ / person per year  Urban location | Rain water recycling for Bangalore City, Bangalore Outer and Mysore. 50% reduction in annual water use rate and water consumption.  We increase urban water demand from 120 (43.8m^3^/year - current estimate) to 150 litres per day (54.75m^3^/year) for scenarios with increased urban water demand. Application of option reduces rate to 27.375m^3^/year. Consumption (amount that is lost from the system) is reduced by 50% to reflect rain water recycling from 30% in current to 15%. |
| Better Enforcement of Laws | % of Unaccounted For Water  Urban locations | Better enforcement of laws is an adaptation option for urban areas. It aims to reduce Unaccounted-For Water (UFW). Reducing UFW is a key aim and an indirect effect of increased cost recovery is a small reduction in urban water demand. For this study we assume an indicative 10% reduction in annual urban water demand and a reduction of consumed water in urban areas (amount that is lost from the system) from the current 30% to 20%, since no reference study is available. |
| Urban Water Pricing | % Urban water demand  Urban locations | Urban water pricing is expected to reduce demand and reduce percentage of water consumed, although no reference studies are available to emulate in the modelling. For this study we assume an indicative 50% reduction in annual urban water demand and reduction in water consumed (amount that is lost from the system) from the current 30% to 20% |
| Increase Micro Irrigation 1.5 mha | Irrigated fraction  Agricultural Locations | Currently, 0.5 mha area is under micro irrigation. So an additional 1.0 mha of micro-irrigation is envisaged with 50% reduction in demand.  From existing irrigated command areas of reservoirs 1.0 mha (10000 sq.km) is converted to micro irrigation and water demand is reduced by 50% compared to present. |
| Increase Micro Irrigation 2.5 mha | Irrigated fraction  Agricultural Locations | Existing 0.5 mha area under micro irrigation. So an addition of 2.0 mha in this option with 50% reduction in demand.  From existing irrigated areas 1.0 mha (100 ha is 1 sq.km, which means 10000 sq.km is converted to micro irrigation) is chosen for reduced water demand by 50% compared to present. All other irrigated catchments in current period and additional irrigated catchments amounting to 10000 sq.km are modelled with reduced water demand by 50%. |
| 5% Drip Irrigation | Irrigated fraction  Agricultural location | Drip Irrigation has a water use efficiency of ~80%. So water demand for the entire irrigated area reduces by 80% of 5%, which is 4%. Change irrigated fraction of historically irrigated catchments to 96% of existing irrigated area.  Accordingly historically irrigated catchments have lower water demand and future irrigation also has lower water demand for all the time steps. Since irrigation increase is interpolated, for each time step (1 year for future) there is an 80% reduction in water demand due to application of 5% drip irrigation. |
| 10% Drip Irrigation | Irrigated fraction  Agricultural location | Change irrigated fraction of historically irrigated catchments to 92% of existing irrigated area. Drip Irrigation has a water use efficiency of ~80% (the same information was given to stakeholders and decision makers). So water demand for the entire irrigated area reduces by 80% of 10%, which is 4%.  Accordingly historically irrigated catchments have lower water demand and future irrigation also has lower water demand for all the time steps. Since irrigation increase is interpolated, for each time step (1 year for future) there is an 80% reduction in water demand due to application of 10% drip irrigation. |
| Agricultural Water Pricing | Irrigated fraction  4 important irrigated areas | Agricultural water pricing is expected to reduce water demand, though no reference studies are available to emulate in the modelling. For this study we assume a 25% reduction in water demand in the irrigated command areas of the four major reservoirs; Krishnaraj Sagar Dam, Harangi Dam, Hemavati Dam and Kabini Dam. |
| Inter-basin Transfer (100 MCM) | Million cubic metre  Transfer over Western Ghats | Transfer from Other Supply node (Netravathy basin to Hemavathy basin) of 100 MCM |
| Inter-basin Transfer (88 MCM) | Million cubic metre  Transfer over Western Ghats | Transfer from Other Supply node (Netravathy basin to Hemavathy basin) of 88 MCM |
| Cauvery Stage V – Phase I (500 MLD) | Million Litres per Day  Bangalore supply | Transmission link from Cauvery river to Bangalore Outer of 500 Million Litres per Day (MLD) |
| Cauvery Stage V – Phase I (270 MLD) | Million Litres per Day  Bangalore supply | Transmission link from Cauvery river to Bangalore Outer of 270 Million Litres per Day (MLD) |
| Urban Lake Restoration | Off-stream reservoir capacity  Bangalore city, Bangalore Outer and Mysore | Urban lakes will improve groundwater recharge and satisfy urban water demand.  In 2001 an area of 105.42 sq.km (Ramesh and Krishnaiah 2013) was covered by lakes in Bangalore. Based on this we assume 100 lakes with a total area 105.42 sq.km, and average depth of 2 metres (assumption) and create an off-stream reservoir in WEAP with corresponding capacity of 210 million cu.m for Bangalore. Proportionately we create a reservoir in Mysore with 10 million cu.m in Mysore because of 5 urban lakes in Mysore.  We model off-stream lake reservoirs by diverting all of the city runoff to these reservoirs. Water available is used to satisfy urban water demand, while excess water runs off to the river. Since about 2/3 area of Bangalore is outside the river basin, we assume that the area outside the basin also contributes to the urban reservoir, and satisfies urban water demand. |
| Urban and Rural Lake Restoration | Off-stream reservoir capacity  Urban and Agricultural Locations | Apart from applying the urban reservoirs described in the urban lake restoration option above, an additional 200 lakes are restored in rural areas.  2 reservoirs are added to the schematic, 1 in Western Ghats catchments and 1 in Non-Western Ghats catchments, each of 210 million cu.m (the same size as the Bangalore reservoir). We divert 10% of the runoff from the respective catchments to the reservoirs, the water from which is used to satisfy irrigation water demand in the respective regions. |

Table S3. Characteristics of adaptations options elicited from stakeholders and details of their incorporation into the water resources model structure

| **Stakeholder type** | **Stakeholder affiliation** | **Role or responsibility** |
| --- | --- | --- |
| Local Government | Bangalore Development Authority | Stakeholder involved in Bangalore city town planning and development |
| Local Government | Bangalore Water Supply and Sewerage Board | City-wide authority with water supply and sewerage management responsibilities |
| State Government | Cauvery Neervari Nigam Ltd. | State government organization involved in decision making in the Cauvery River Basin in Karnataka |
| State Government | Karnataka Urban Water Supply and Drainage Board | State government implementing body for water supply and drainage schemes in urban areas except Bangalore city |
| State Government | Department of Agriculture | Stakeholder involved in regional agricultural water management issues |
| National Government | Central Ground Water Board | Research on and supporting the management of state groundwater resources |
| Non-Governmental Organization | Ashoka Trust for Research in Ecology and the Environment | Research, advocacy and practice in environmental issues, including water resources management and socio-ecology |
| Non-Governmental Organization | Centre for Study of Science, Technology and Policy | Supporting governmental policy and decision-making in various sectors including energy and water resources |
| University academic | Indian Institute of Science | Part of a nodal agency between academia and government on water resources planning and management |

Table S4. Table of affiliations and roles/responsibilities of stakeholders involved in the workshops.
